# Energy Efficiency Maximization for Multi-Cell Multi-Carrier NOMA Networks

**DOI:** 10.3390/s20226642

**Published:** 2020-11-20

**Authors:** Abuzar B. M. Adam, Xiaoyu Wan, Zhengqiang Wang

**Affiliations:** School of Communication and Information Engineering, Chongqing University of Posts and Telecommunications, Chongqing 400065, China; l201810010@stu.cqupt.edu.cn (A.B.M.A.); wanxy@cqupt.edu.cn (X.W.)

**Keywords:** energy efficiency, fractional programming, non-orthogonal multiple access, quadratic transform, user association

## Abstract

As energy efficiency (EE) is a key performance indicator for the future wireless network, it has become a significant research field in communication networks. In this paper, we consider multi-cell multi-carrier non-orthogonal multiple access (MCMC-NOMA) networks and investigate the EE maximization problem. As the EE maximization is a mixed-integer nonlinear programming NP-hard problem, it is difficult to solve directly by traditional optimization such as convex optimization. To handle the EE maximization problem, we decouple it into two subproblems. The first subproblem is user association, where we design a matching-based framework to perform the user association and the subcarriers’ assignment. The second subproblem is the power allocation problem for each user to maximize the EE of the systems. Since the EE maximization problem is still non-convex with respect to the power domain, we propose a two stage quadratic transform with both a single ratio quadratic and multidimensional quadratic transform to convert it into an equivalent convex optimization problem. The power allocation is obtained by iteratively solving the convex problem. Finally, the numerical results demonstrate that the proposed method could achieve better EE compared to existing approaches for non-orthogonal multiple access (NOMA) and considerably outperforms the fractional transmit power control (FTPC) scheme for orthogonal multiple access (OMA).

## 1. Introduction

As the largest sector in information and communication technology, wireless networks have been developed quite rapidly since the first-generation (1G) to the fourth-generation (4G) to support the need for higher communication capacity and the explosive growth of the number of users. However, even 4G with its high mount of data has shown lags in supporting such needs [1,2]. Therefore, next-generation networks should be designed to meet these increasing needs to satisfy the growing demand for high data rates [3]. This led to the investigation of fifth-generation (5G) mobile communication to enhance system capabilities [1,4,5,6]. Consequently, energy consumption will be a significant issue in 5G since it leads to environmental and economic problems [1]. Hence, green communication networks have become a promising research field [7,8].

Non-orthogonal multiple access (NOMA) has been considered as a promising technology for 5G. Unlike orthogonal multiple access (OMA), NOMA provides high spectral efficiency (SE) by allowing more than one user to be multiplexed simultaneously in the same subcarrier with different power levels. Simultaneously, successive interference cancellation (SIC) is exploited at the receiver to deal with the inevitable interference within the same subcarrier [9,10]. In order for SIC to work, we have to deal with the inevitable interference within the same subcarrier [9,10]. For SIC to work appropriately in extracting the desired signal, users’ power must be allocated properly and efficiently. As a consequence, many studies have considered studying NOMA in the power domain recently [11,12,13].

Many works have investigated energy efficiency (EE) for orthogonal frequency division multiple access (OFDMA) [14,15,16,17,18,19], but it has shown lags behind NOMA in achieving higher SE and EE. Various topologies of NOMA have been investigated in many studies with regard to EE.

EE for a single cell topology was investigated in [20,21]. In [20], the authors considered solving EE of the network whereby each user has its minimum rate constraint, as well as minimum power. The authors in [21] proposed power optimization for each user separately. Then, the EE problem was solved to find the optimal power. Several works have been introduced in multi-carrier NOMA (MC-NOMA) networks. In these networks, the power allocation and subchannels were jointly optimized [22,23]. Thus, in [24], user resource block (user-RB) association and power allocation were jointly studied for uplink hybrid NOMA-OMA networks. An EE maximization problem was formulated to represent user clustering, channel assignment, and power allocation. Graph theory and swap matching were used to construct the final algorithm. Joint power and bandwidth allocation were investigated in [25] to maximize system EE under the base station (BS) power and user’s minimum rate requirement. In [26], the authors considered maximizing minimum individual EE of users to guarantee fairness between users. In [27], the authors proposed energy efficient subchannel assignment and the power proportional factor calculation method for multiplexed users. In [28], joint resource management of user clustering and power allocation were investigated for EE maximization in hybrid NOMA systems. User clustering was used to convert the mixed integer non-linear problem into tractable equivalent problems. The optimal power allocation solution was found for limited power consumption and full power consumption through the iterative solution of the equivalent problems.

Game theory was efficiently used in resource allocation problems for NOMA [29,30,31]. In [29], the EE problem was decoupled into subchannel assignment, which was solved by matching theory, and power allocation, which was solved by a super-modular game. Finally, the EE problem was solved using successive convex approximation (SCA). The study in [30] presented an optimization technique to improve both SE and EE while satisfying users’ quality of service (QoS) constraint and transmit power budget. A joint SE and EE was formulated, then the dual decomposition technique was used to find the optimal solution. To achieve the SE and EE tradeoff in hybrid MC-NOMA, resource allocation with minimum rate requirements was investigated in [31]. Access mode selection, resource allocation, subchannel assignment, and users’ clustering were jointly considered. The SE-EE problem was formulated as a multi-objective optimization problem and converted to single-objective optimization. Then, Lagrangian dual decomposition and sequential convex programming were used to solve the latter problem.

In practice, wireless communications systems are usually multi-cell systems. Resource management has been addressed from different perspectives for multi-cell NOMA [32,33,34,35,36,37,38,39,40]. Resource optimization was studied in [32] to achieve the optimal resource utilization by optimizing power allocation and the user pair. Since the intercell topology inherits intercell interference (ICI), the authors in [33] used the proportional relation between the load and the interference among cells to optimize power allocation and thus energy minimization. In order to maximize the minimum throughput, the authors in [34] addressed time and power allocation optimization for wireless powered networks. Power allocation was handled by geometric programming, while the Hopfinger golden section search method was used to find optimal time allocation. The authors in [35] investigated the resource management problem in multi-cell multi-carrier NOMA (MCMC-NOMA) networks with the quality of experience (QoE). The resource allocation problem was formulated as sum mean opinion scores (MOSs). Hence, this problem was decoupled into two subproblems where the relation among users’ BSs and subchannels was handled by 3D matching. On the other hand, the optimal power allocation problem was solved using continuous power allocation and SCA. The authors in [36] investigated EE in MCMC-NOMA networks using the non-cooperative auction-based game for power allocation for users and graph-based k-means clustering to mitigate the inter-cell interference. Moreover, transmit power minimization in MCMC-NOMA networks was considered in [37]. Centralized minimum power control for fixed user assignment was exploited, then the greedy user clustering and power allocation scheme was proposed. Efficient power allocation was proposed in [38] to maximize system sum capacity under the user required capacity constraint. The optimal power allocation was obtained iteratively via local optimal solution where Karush–Kuhn–Tucker conditions were satisfied. To maximize EE and minimize transmission power in the presence of ICI, the authors in [39] first studied joint optimization of the subchannel and power allocation. Then, the fuzzy logic-based multiple criteria were used to balance among user’s received signal and the level of interference to allocate resources to users in NOMA coordinated systems jointly. In [40], power control to maximize the sum rate in multi-cell NOMA networks was introduced. First, a single cell power allocation problem was considered, then power control in multiple cells was used to obtain the optimal solution, as well as achieving high EE. The authors in [41] investigated the power control in multi-cell NOMA systems. The authors formulated the main problem as power minimization subproblems. Then, a distributed algorithm was proposed to handle the problem. In [42], the joint user grouping, beamforming (BF), and power control problem was studied. The beamforming was performed for each cell locally using the zero-forcing beamforming technique. Then, two user grouping strategies were proposed, which were independent of the power control.

The objective of this paper is to study the joint user association and power allocation for EE maximization in MCMC-NOMA networks. In order to properly handle EE maximization in MCMC-NOMA networks, user association and resource allocation to satisfy user’s needs (i.e., the QoS requirement) should be dealt with. Consequently, the problem tends toward having more difficulties, and our objective is to deal with those issues. The contributions of this paper can be summarized as follows:

- Different from [36,38], we investigate the joint resource allocation for downlink MCMC-NOMA networks. The EE of the network is defined as a sum of energy efficiencies of users in each BS. The problem is formulated as a mixed integer non-convex optimization problem with constraints including the minimum power for each user, the maximum sum power consumption for users in a cell, the minimum rate for each user and user association, as well as number of available subcarriers for each user, where each user can associate with one BS via one subcarrier.
- We decouple the optimization problem into two subproblems. The first problem is the user association and subcarrier assignment where we design a matching-based algorithm to handle it. The second subproblem is the power allocation for EE maximization. Although power allocation across multiple interfering links is challenging, in the proposed framework, we employ the multidimensional quadratic transform to convert the non-convex problem into an equivalent tractable sequence of convex problems by decoupling the signal and the interference terms in the signal-to-interference-plus-noise ratio (SINR). The final equivalent problem can be solved by a standard optimization method, and it is guaranteed to converge to a stationary point of the original problem.
- For further verification and performance evaluation, the proposed method is extensively tested through simulation. Despite the relatively slow convergence of the proposed power allocation method, this gives the proposed method the capability of exploring all the possible solutions. The simulation results show that the proposed approach achieves better performance than other NOMA-based schemes, as well as OFDMA systems.

The rest of this paper is organized as follows. In Section 2, we introduce the system model and the problem formulation of EE maximization. In Section 3, we present the proposed framework, which includes user association and power allocation by solving the EE maximization problem. In Section 4, we evaluate the performance of our proposed method and compare it with other existing works using simulation. Finally, conclusions and future work are presented in Section 5.

All the adopted notations used in this paper are included in Table 1.

## 2. System Model and Problem Formulation

### 2.1. MCMC-NOMA Network

Consider a downlink MCMC-NOMA network with *M* cells communicating with *N* users through *K* subcarriers, denoted as M={1,…,M}, N={1,.…,N} and K={1,.…,K}. Each cell has one BS in the center with maximum power $P_{m}$. We consider the case of the ICI. Assume that each BS shares the same spectrum where the total bandwidth *W* is divided equally into *K* subcarriers and given as Wk=WK. Unlike OMA, those subcarriers can be concurrently accessed by multiple users. Suppose that the set of users served by BS *m* on subcarrier *k* is denoted by $N_{m,k}$, then the channel coefficient between the BS *m* and the user $i\in N_{m,k}$ is given by:

(1) $$h_{m,i,k}=g_{m,i,k}d_{m,i}^{-\alpha }$$

where $g_{m,i,k}$ is the fading channel gain between the user *i* and the serving BS *m* on subcarrier *k* assuming this channel fading follows a Rayleigh distribution. $d_{m,i}$ is the distance between the user *i* and BS *m*. α is the path loss exponent. Without loss of generality, the following order can be assumed:

(2) $$\left|h_{m,1,k}\right|\leq \left|h_{m,2,k}\right|\leq \ldots \leq \left|h_{m,N_{m,k},k}\right|,\forall m\in M,k\in K$$

### 2.2. Interference Modeling in MCMC-NOMA Networks

In MCMC-NOMA networks, two types of interference are present, intra-cell and ICI. During the SIC process, ICI is treated as noise, while the intra-cell interference is partially canceled by SIC [43]. Consequently, the decoding order of the users in the MCMC network is highly impacted by the ICI [44,45].

According to the NOMA protocol, the BS can deliver a superposition signal to its users, which can be given as:

(3) $$x_{m,k}=\sum_{i=1}^{N}\gamma_{m,i,k}\sqrt{p_{m,i,k}}x_{m,i,k}$$

where $\gamma_{m,i,k}$ is the user assignment indicator. $\gamma_{m,i,k}=1$ indicates the user *i* is served by BS *m* on subcarrier *k* and $\gamma_{m,i,k}=0$ if otherwise. $p_{m,i,k}$ is the allocated power of user *i* on subcarrier *k*, and $x_{m,i,k}$ is the desired signal.

Since user *i* receives interference from other users assigned to the same subcarrier, then the received signal by user *i* covered by BS *m* is given by:

(4) $$\begin{array}{cc} y_{m,i,k} & =h_{m,i,k}x_{m,k}+\sum_{n=1,n\neq m}^{M}h_{n,i,k}x_{n,k}+n_{i,k}\\ \; & =\sum_{i=1}^{N}\gamma_{m,i,k}h_{m,i,k}\sqrt{p_{m,i,k}}x_{m,i,k}\\ \; & +\sum_{n=1,n\neq m}^{M}\sum_{i=1}^{N}h_{n,i,k}\sqrt{p_{n,i,k}}x_{n,i,k}+n_{i,k} \end{array}$$

where $h_{m,i,k}$ is the cross-channel coefficient between BS *n* and user *i* served by BS *m* on the subcarrier *k*. $n_{i,k}$ is the additive white Gaussian noise with zero mean and variance $\sigma^{2}$. Based on the NOMA principle, each user should decode the users in the same cell with weaker channel coefficients before detecting its own message. Recalling back the effect of ICI, we can assume the following order:

(5) $$\left|H_{m,1,k}\right|\leq \left|H_{m,2,k}\right|\leq ..\ldots \ldots \leq \left|H_{m,N_{m,k},k}\right|$$

where,

(6) $$H_{m,i,k}=\frac{{\left|h_{m,i,k}\right|}^{2}}{\sigma^{2}+\sum_{n\neq m}^{M}P_{n,k}{\left|h_{n,i,k}\right|}^{2}}$$

where $P_{n,k}=\sum_{i=1}^{N_{n,k}}p_{n,i,k}$ is the total transmission power of BS *n* over subcarrier *k*. Thus, the received SINR for user *i* to detect user *l*’s signal on the subcarrier *k*, l≤i, can be expressed as:

(7) $$\Gamma_{m,i,l,k}=\frac{\gamma_{m,i,k}{\left|h_{m,i,k}\right|}^{2}p_{m,l,k}}{\sigma^{2}+\sum_{q=l+1}^{N}\gamma_{m,i,k}{\left|h_{m,i,k}\right|}^{2}p_{m,q,k}+\sum_{n\neq m}^{M}P_{n,k}{\left|h_{n,i,k}\right|}^{2}}$$

If user *l*’s message is detected, then the received SINR at user *i*’s receiver to detect its own message is given as:

(8) $$\Gamma_{m,i,k}=\frac{\gamma_{m,i,k}{\left|h_{m,i,k}\right|}^{2}p_{m,i,k}}{\sigma^{2}+\sum_{j=i+1}^{N}\gamma_{m,i,k}{\left|h_{m,i,k}\right|}^{2}p_{m,j,k}+\sum_{n\neq m}^{M}P_{n,k}{\left|h_{n,i,k}\right|}^{2}}$$

The achievable data rate of user *i* served by BS *m* on the subcarrier *k* can be expressed by:

(9) $$R_{m,i,k}=W_{k}\log_{2}\left(1+\Gamma_{m,i,k}\right)$$

The EE for each user is defined as the user achievable rate divided by its total assigned power, and thus:

(10) $$EE_{m,i,k}=\frac{R_{m,i,k}}{p_{m,i,k}+p_{c}}$$

where $p_{c}$ is the circuit power consumption. Then, we can obtain the total EE of the network by the following (see [46,47]):

(11) $$EE=\sum_{m=1}^{M}\sum_{i=1}^{N}\sum_{k=1}^{K}EE_{m,i,k}$$

### 2.3. Problem Formulation

Our goal is to maximize the EE for the network under the minimum data rate and maximum power consumption constraints. Then, the optimization problem of EE maximization can be given as:

(12) $$\begin{array}{cccc} \; & \max_{\gamma ,p}\; & \; & EE\\ \; & s.t.\; & \; & C1:\sum_{i=1}^{N}\sum_{k=1}^{K}\gamma_{m,i,k}p_{m,i,k}\leq P_{m},\forall m\in M,\\ \; & \; & & C2:p_{m,i,k}\geq 0,\forall m\in M,i\in N,k\in K,\\ \; & \; & & C3:R_{m,i,k}\geq R_{\min},\forall m\in M,i\in N,k\in K,\\ \; & \; & & C4:H_{m,i,k}\leq H_{m,j,k},\forall m\in M,\left(i,j\right)\in N,k\in K,\\ \; & \; & & C5:\gamma_{m,i,k}\in \left\{0,1\right\},\forall m\in M,i\in N,k\in K,\\ \; & \; & & C6:\sum_{m=1}^{M}\sum_{k=1}^{K}\gamma_{m,i,k}\leq 1,i\in N, \end{array}$$

Constraint *C*1 denotes that the total power consumption of the multiplexed users in cell *m* should be less than the maximum power of the BS. Constraint *C*2 indicates that the user’s allocated power should be non-negative. Constraint *C*3 implies the minimum rate constraint for the cell *M*. *C*4 is to guarantee the successful implementation of SIC in a specific order [35]. Constraints *C*5 and *C*6 ensure that each user is associated with one BS and occupies one subcarrier at most. Since the EE maximization problem for single cell MC-NOMA is NP-hard, as discussed in [29], obtaining the corresponding power to maximize the EE in each cell actually depends on the changes of the allocated power in other cells due to the presence of the ICI. Due to the above reasons, the problem above is mixed integer nonlinear programming (MINLP), which is an NP-hard problem. Next, we propose a method by joint user association and power allocation to find an efficient solution to (12).

## 3. Proposed Method

Since the relevant problem in Equation (12) is non-convex and NP-hard, it is difficult to find the optimal solution for this problem. Therefore, we decompose the problem into two subproblems to give an efficient algorithm. The first subproblem is the user association problem where we design a matching-based algorithm to handle it. After we obtain the user association, then we give an efficient algorithm to find the power allocation for the EE maximization problem based on the quadratic transform method with a fixed user association obtained by the first subproblem.

### 3.1. Matching Scheme for User Association and Subcarrier Assignment

In this subsection, we design the matching-based user association scheme as [29,35,48]. Mathematically, the user association subproblem can be defined as:

(13) $$\begin{array}{cccc} \; & \max_{\gamma }\; & \; & EE\\ \; & s.t.\; & \; & C3,C5,C6, \end{array}$$

The scheme has two steps, which are given in Algorithm 1. The first step is to associate the users with the BSs. The second step is to assign the subcarriers to the users in each cell as in Algorithm 2. To compare the EE of all the users in each subcarrier for a given cell, we allocate initial power to the users such that Constraint C3 is fulfilled. Hence,

(14) $$p_{m,i,k}=\frac{2^{\frac{KR_{\min}}{W}}-1}{{\left|h_{m,i,k}\right|}^{2}}$$

**Algorithm 1** User association matching scheme.1:Initialize the power for all users using (14)2:Initialize the associated users AU, unassociated users $UU={\left[\left|h_{m,i}\right|\right]}_{M\times N}$, and UB lists to record the associated users with the BSs, the unassociated users, and the preferable BS, respectively.3:**while **UU≠ϕ** do**4:
 **for** i=1:N **do**
5:
  **if** i∈UU **then**
6:   Find the maximum value $\left|h_{m,i}\right|$ in UU according to:
(15)$$\left|h_{m,i}\right|=\arg\max UU_{M\times N}$$7:   Assign the user to the BS m from the list UB, and remove him/her from UU.8:   **if** all BSs have more than one user, calculate the EE $EE\left(\pi_{m}\right),m=1,\ldots ,M$ for all possible $\pi_{m}=\left\{AU\left(m\right),\left\{i\right\}\right\}$ sets. **then**9:    Select the set that has maximal EE:
(16)$\pi =\underset{\pi_{m}}{\arg\max}EE\left(\pi_{m}\right)$10:    Update AU by adding the new set π. Update UUby removing the elements of the set π.11:    Update UB by removing the preference *m* of the unmatched user.12:**   end if**13:**  end if**14:** end for**15:**end while**

**Algorithm 2** Subcarrier assignment matching scheme.
Initialize power allocation for all users.Obtain users’ BSs association by Algorithm 1.Initialize the matched MU, the unmatched $UMU={\left[\left|h_{m,i,k}\right|\right]}_{N\times K}$ for a given cell *m*, and UK lists to record the matched users, the unmatched, and the user’s preferable subcarrier.
**while** UMU≠ϕ **do**

 **for** i=1:N **do**

  **if** i∈UMU **then**
   Find the maximum value $\left|h_{m,i}\right|$ from UMU according to:
(17)$$\left|h_{m,i,k}\right|=\underset{i\in AU}{\arg\max}UMU_{N\times K}$$   Match the user *i* with the subcarrier *k*, and remove him/her from UMU.   Assume each subcarrier *k* has a matching user *i*; we have $T_{u}=\left\{MU\left(k\right),\left\{i\right\}\right\},u=1,..\ldots ,K$   
**if** $MU\left(k\right)\geq 1$ **then**
    Calculate the EE, and select the set with the higher EE such that:
(18)$T=\underset{T_{u}}{\arg\max}EE\left(T_{u}\right)$    Update MU, and remove the selected users from UMU. Update UK by removing the matched subchannel.
**   end if**

**  end if**

** end for**

**end while**



The UU list is initialized to record the users who have not been associated with any BS. At the beginning of the association procedure, we assume the users can use all subchannels; the users with maximal channel coefficients are matched first with the BSs, then other users are selected by the BS only if they can provide a higher EE. The associated users are removed from the UU list and added to the AU list, which includes the associated users. We repeat the process until the UU list is empty.

When we obtain cell association, then we define MU, UMU, and UK to record the matched users, the users who have not assigned been subcarriers, and the user’s preferable subcarrier. Similar to the cell association, we first find the users with the maximal channel coefficients and assign them the subcarriers. Then, we update the UMU by removing the selected users. MU is updated by adding the updated users. For other users, the subcarrier will select the user who will provide higher EE. The UK list is updated by removing the matched users and emptied if all subcarriers are assigned to users.

In Algorithm 1, initially, each user selects one BS, and each BS accepts multiple users. Assume the worst case of the number of users that select the BSs is *N*. Then, the complexity of this step is $O\left(M^{2}N\right)$. For the update step, the BS accepts the user that achieves higher EE. Let $T_{t}$ denotes the number of iterations. The complexity of the update step is $O\left(NMT_{t}\right)$. The complexity of Algorithm 1 is $O\left(NM\left(M+T_{t}\right)\right)$.

For Algorithm 2, in the initialization step, the subcarrier accepts multiple tuples of users and BSs. The complexity of this step is $O\left(M^{2}K^{2}\right)$. In the update step, each subcarrier admits the number of tuples that can achieve higher EE. Let $T_{t}^{\prime }$ denote the number of iterations. The complexity of this step in the worst case is $O\left(T_{t}^{\prime }MNK\right)$. The complexity of Algorithm 2 is $O\left(MK\left(MK+NT_{t}^{\prime }\right)\right)$.

### 3.2. Energy-Efficient Power Allocation

Since user association has been solved, now, our EE problem can be transformed to the optimization problem as a function of power (i.e., EE can be improved by optimizing the transmit power in each user). Even for user association and subcarrier assignment obtained by Algorithms 1 and 2, respectively, it is difficult to find the optimal power allocation for (12) because it is the sum of the ratio problem, which is NP-hard. To find the efficient power allocation, we use the quadratic transform method to solve our problem by fractional programming (FP) [49]. Our power allocation subproblem is equivalent to:

(19) $$\begin{array}{cccc} \; & \max_{p}\; & \; & f\left(p\right)\\ \; & s.t.\; & \; & C1-C4 \end{array}$$

Primarily, the multidimensional quadratic transform is exploited to address the problem starting with the single ratio quadratic transform user’s EE. Thus,

(20) $$f_{q}\left(p,\varsigma \right)=\sum_{m=1}^{M}\sum_{i=1}^{N}\sum_{k=1}^{K}2\varsigma_{m,i,k}{\left(R_{m,i,k}\right)}^{\frac{1}{2}}-\varsigma_{m,i,k}^{2}\left(p_{m,i,k}+p_{c}\right)$$

Substituting (20) into (19), the term ${\left(R_{m,i,k}\right)}^{2}$ is non-decreasing, and the term $p_{m,i,k}+p_{c}$ is concave. To recast the term $\left(R_{m,i,k}\right)$ as concave, let $R\left(p,\Gamma \right)$ be the equivalent function of the user’s data rate $R_{m,i,k}$ in (20). Recasting $R\left(p,\Gamma \right)$ using the closed-form FP approach [49], we get:

(21) $$R^{CF}\left(p,\Gamma \right)=\log_{2}\left(1+\Gamma_{m,i,k}\right)-\Gamma_{m,i,k}+\frac{\left(1+\Gamma_{m,i,k}\right){\left|h_{m,i,k}\right|}^{2}p_{m,i,k}}{\sigma^{2}+\sum_{j=1,j\neq i}^{N}{\left|h_{m,i,k}\right|}^{2}p_{m,j,k}+\sum_{n\neq m}P_{n,k}{\left|h_{n,i,k}\right|}^{2}}$$

where the optimal value for $\Gamma_{m,i,k}^{*}$ is calculated when $p_{m,i,k}$ is fixed as follows:

(22) $$\Gamma_{m,i,k}^{*}=\frac{{\left|h_{m,i,k}\right|}^{2}p_{m,i,k}}{\sigma^{2}+\sum_{j=1,j\neq i}^{N}{\left|h_{m,i,k}\right|}^{2}p_{m,j,k}+\sum_{n\neq m}P_{n,k}{\left|h_{n,i,k}\right|}^{2}}$$

Since $R^{CF}\left(p,\Gamma \right)$ still contains ratio forms, we continue with our multidimensional quadratic transform of the data rate function, and that yields equation:

(23) $$\begin{array}{cc} R_{q}^{CF}\left(p,\Gamma ,y\right)= & 2y_{m,i,k}{\left(\left(1+\Gamma_{m,i,k}\right){\left|h_{m,i,k}\right|}^{2}p_{m,i,k}\right)}^{\frac{1}{2}}\\ \; & -y_{m,i,k}^{2}\left(\sigma^{2}+I_{m,i,k}\right)+\Gamma^{const} \end{array}$$

where $I_{m,i,k}$ is the aggregated interference experienced by the user *i* and $\Gamma^{const}$ refers to a constant term when Γ is fixed. The term ${\left(\left(1+\Gamma_{m,i,k}\right){\left|h_{m,i,k}\right|}^{2}p_{m,i,k}\right)}^{\frac{1}{2}}$ is concave, and $R_{q}^{CF}\left(p,\Gamma ,y\right)$ is concave as well. The optimal $y_{m,i,k}$ is given by:

(24) $$y_{m,i,k}^{*}=\frac{{\left(\left(1+\Gamma_{m,i,k}\right){\left|h_{m,i,k}\right|}^{2}p_{m,i,k}\right)}^{\frac{1}{2}}}{\sigma^{2}+\sum_{j=1,j\neq i}^{N}{\left|h_{m,i,k}\right|}^{2}p_{m,j,k}+\sum_{n\neq m}P_{n,k}{\left|h_{n,i,k}\right|}^{2}}$$

Substituting (23) into (20), we get:

(25) $$\begin{array}{cc} f_{qq}^{CF}\left(p,\varsigma ,\Gamma ,y\right)= & \sum_{m=1}^{M}\sum_{i=1}^{N}\sum_{k=1}^{K}2\varsigma_{m,i,k}{\left(R_{q}^{CF}\left(p,\Gamma ,y\right)\right)}^{\frac{1}{2}}\\ \; & -\varsigma_{m,i,k}^{2}\left(p_{m,i,k}+p_{c}\right) \end{array}$$

Hence, the power allocation problem for Problem (19) with fixed user association can be equivalent to the following problem by quadratic transform in [49].

(26) $$\begin{array}{cccc} \; & \max_{p,\varsigma ,\Gamma ,y}\; & \; & f_{qq}^{CF}\left(p,\varsigma ,\Gamma ,y\right)\\ \; & s.t.\; & \; & C1:\sum_{i=1}^{N}p_{m,i,k}\leq P_{m}\\ \; & \; & & C2:p_{m,i,k}\geq 0\\ \; & \; & & C3:R_{q}^{CF}\left(p,\Gamma ,y\right)\geq R_{\min}\\ \; & \; & & C4:H_{m,i,k}\leq H_{m,j,k}\\ \; & \; & & C5:\varsigma_{m,i,k}\in R\\ \; & \; & & C6:\Gamma_{m,i,k}\in R\\ \; & \; & & C7:y_{m,i,k}\in R \end{array}$$

When other variables are fixed, the optimal value of the auxiliary variable $\varsigma_{m,i,k}$ is given by:

(27) $$\varsigma_{m,i,k}^{*}=\frac{{\left(R_{m,i,k}\right)}^{\frac{1}{2}}}{p_{m,i,k}+p_{c}}$$

The convexity of Problem (26) is attained when both auxiliary variables $y_{m,i,k}$ and $\varsigma_{m,i,k}$ are fixed; consequently, classical numerical methods such as the interior point method [50] can be used to find the optimal solution for (26).

CVXis a popular system used for solving and constructing disciplined convex programs [51], since it supports a wide spread of standard optimization problems, and it is used to model and solve Problem (26). The power allocation can be obtained iteratively as in Algorithm 3. Algorithm 4 summarizes the EE maximization scheme for the MCMC-NOMA network. The convergence of Algorithm 3 is achieved in iterations O(log(1ϵ)), where ϵ is an error tolerance (see Table 2).
**Algorithm 3** Power allocation for EE maximization.**Initialization: **$R_{min},t=0,p_{m,i,n},\epsilon$** while**$\left|f_{qq}^{CF}\left(t+1\right)-f_{qq}^{CF}\left(t\right)\right|>\epsilon$** do****  for **m=1:M** do****   for**k=1:K**do**    Determine the decoding order.    Update $\Gamma_{m,i,k}$ by (22) with fixed $p_{m,i,k}$.    Update $y_{m,i,k}$ by (24).    Update $\varsigma_{m,i,k}$ by (27).    Update $p_{m,i,k}$ by solving (26) for fixed $y_{m,i,k}$ and $\varsigma_{m,i,k}$.**  end for**** end for****end while****Algorithm 4** Joint user association and power allocation for EE maximization.1:Obtain user association and subcarrier assignment by Algorithms 1 and 2, respectively.2:Calculate the power allocation using Algorithm 3.3:Compute the EE according to (11).

To verify that Algorithm 3 converges to the stationary point of (19), first, we take into consideration that $f\left(p\right)=\sum_{m=1}^{M}\sum_{i=1}^{N}\sum_{k=1}^{K}EE_{m,i,k}$ where $EE_{m,i,k}$ is the non-decreasing sum of the functions of the ratio. Problem (19) is equivalent to:

(28) $$\begin{array}{cccc} \; & \max_{p,\varsigma }\; & \; & f_{q}\left(p,\varsigma \right)\\ \; & s.t.\; & \; & C1-C4\\ \; & \; & & C5:\varsigma \in R \end{array}$$

Second, we rewrite Problem (19) as a function of EE; hence:

(29) $$\begin{array}{cccc} \; & \max_{p,EE}\; & \; & f\left(EE\right)\\ \; & s.t.\; & \; & C1-C4\\ \; & \; & & E_{m,i,k}=\frac{R_{m,i,k}}{p_{m,i,k}+p_{c}} \end{array}$$

Then, according to the quadratic transform in [49], the variable $EE_{m,i,k}$ can be replaced by $2\varsigma_{m,i,k}{\left(R_{m,i,k}\right)}^{\frac{1}{2}}-\varsigma_{m,i,k}^{2}\left(p_{m,i,k}+p_{c}\right)$. Since *f* is a non-decreasing function, $\max_{p}\sum_{m=1}^{M}\sum_{i=1}^{N}\sum_{k=1}^{K}\max_{\varsigma }\left(2\varsigma_{m,i,k}{\left(R_{m,i,k}\right)}^{\frac{1}{2}}-\varsigma_{m,i,k}^{2}\left(p_{m,i,k}+p_{c}\right)\right)$ can be rewritten as (28).

After recasting the rate term as concave as in (23), Problem (26) is obtained, when $p_{m,i,k}$ and $\varsigma_{m,i,k}^{*}$ are fixed; representing the stationary point at which Algorithm 3 converges.

## 4. Simulation Results

In this section, we present our simulation results to evaluate the performance of the proposed method for MCMC-NOMA network. Through this simulation, each cell consists of one BS in the center with a radius of 500 m. All cells have an equal number of users, and the users are distributed randomly in the cell region. The total bandwidth of the system is 5 MHz divided equally by *K* subcarriers (the bandwidth is divided into 20 subcarriers). The noise power spectral density is −174 dBm/Hz. For the propagation model, we assume there is small-scale fading, shadow fading, and distance dependence path loss. For small-scale fading, the subcarrier signal of each user experiences Rayleigh fading, and the log-normal shadowing has a standard deviation of 8 dB. The distance dependent path loss is assumed 128.1 + 37.6log${}_{10}$d dB, where the distance between the user and the BS is in km. Table 2 illustrates the simulation parameters.

For the sake of performance comparison, the three step DGA (specifically, the projected gradient descent algorithm) [52] is used with our constraints. For NOMA fractional transmit power allocation (NOMA-FTPA) [53], we use the same reasoning for the user association in our algorithm to find the EE and the decay factor is set to 0.2. For the comparison with OMA, the fractional transmit power control scheme (OMA-FTPC) [54,55] is invoked to multiplex multiple users in the same subcarrier through time slots.

**Figure 1:**
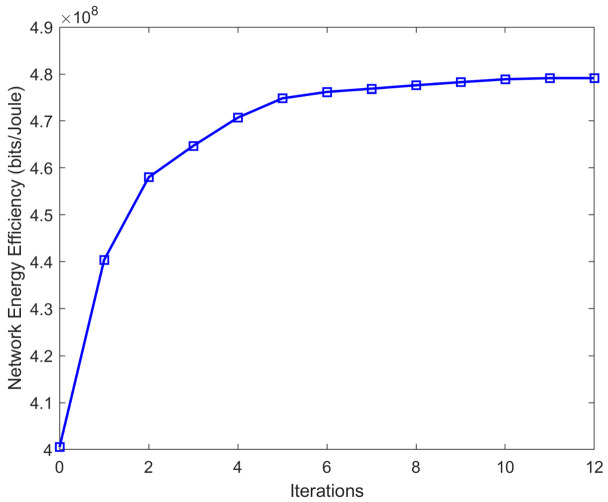
EE versus the number of iterations.

First, we evaluate the performance of our proposed framework with regard to the number of iterations. Setting the number of users to 10 per BS, the number of BSs to 10, $P_{m}$ is set to 10 W, and $p_{c}=0.5$ W. Figure 1 shows the EE of the network obtained by our algorithm versus the number of iterations. One can observe that the EE increases with each iteration and reaches the convergence point in a few iterations. Despite the relatively slower convergence speed of the proposed method compared to the conventional FP methods, the proposed method has the advantage of exploring all of the solution space.

**Figure 2:**
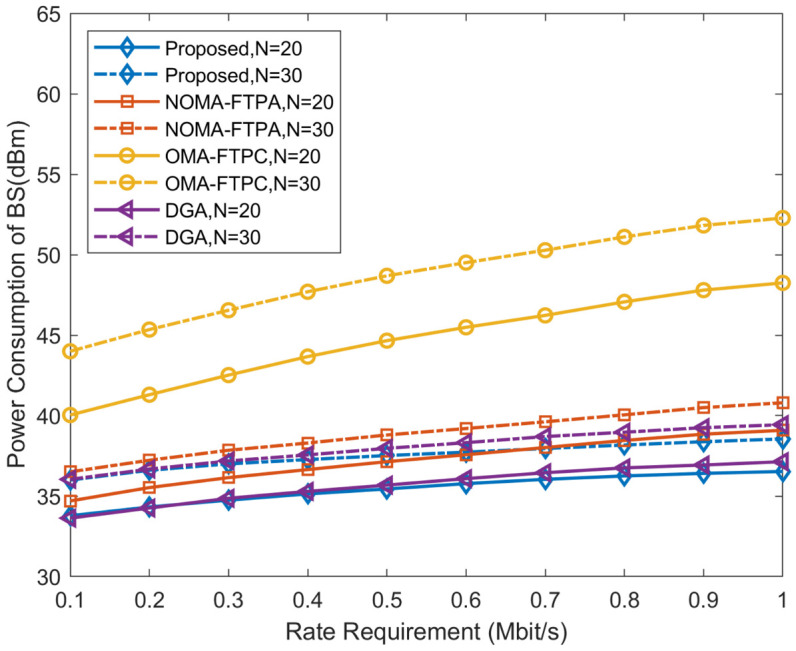
Power consumption for different rate requirements.

**Figure 3:**
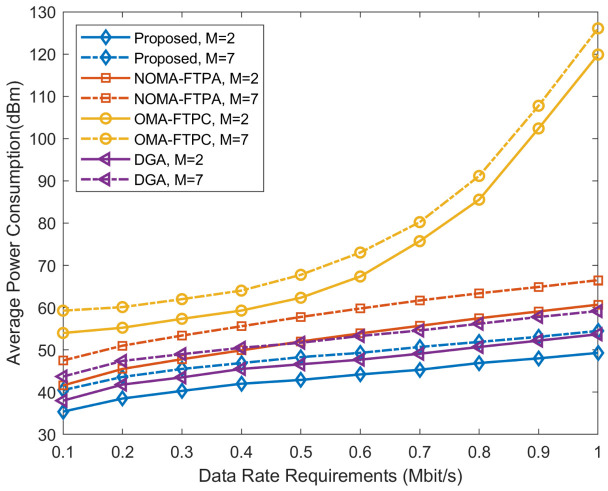
Power consumption for different numbers of BSs versus different data rate requirements.

Figure 2 and Figure 3 illustrate the performance comparison with regard to the required data rate and corresponding power consumption to achieve that rate. In Figure 2, using similar values for the parameters, it is clear to observe that the proposed algorithm can achieve better performance in consuming lower power to reach the required rate followed by the DGA and NOMA-FTPA. OMA-FTPC has the worst performance among the four methods. It can be noticed that the increase in the number of users leads to higher power consumption for the sake of offsetting the ICI and fulfilling the data rate requirements of the users.

In Figure 3, different numbers of BSs are simulated to attain more evaluation of the performance in the multi-cell multi-carrier topology with a fixed number of users per BS, and the maximum power of each BS station is set to 10 Watts. As can be noticed, the proposed algorithm still dramatically outperforms other approaches followed by the DGA and NOMA-FTPA, then at the end, OMA-FTPC. For rate requirement of 0.1 Mbit/s and m=7, the power consumptions are 40.5 dBm, 43 dBm, 44.51 dBm, and 59.3 dBm for the proposed method, DGA, NOMA-FTPA, and OMA-FTPC, respectively. The drastic increment of the power consumption with the increasing number of BSs is to overcome the severe influence of the ICI and strive to fulfill each user’s data rate requirement.

**Figure 4:**
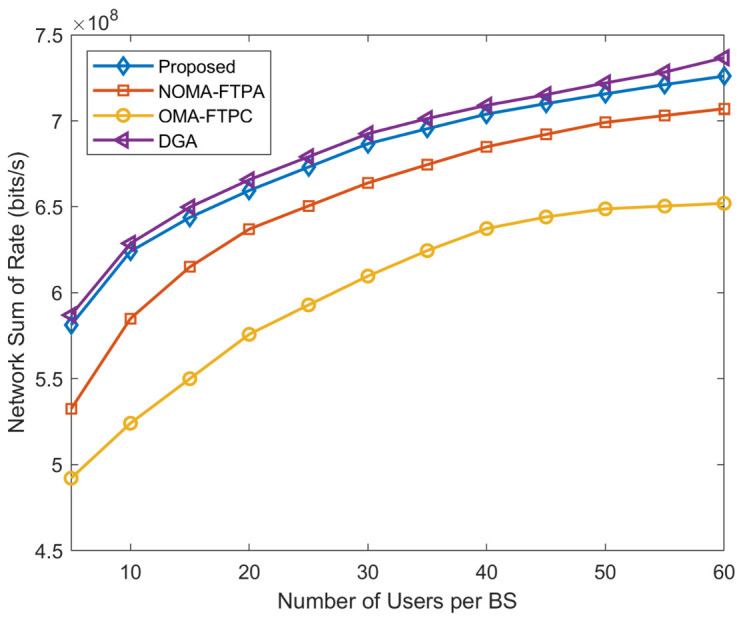
Network sum rate with different numbers of users per BS.

Figure 4 demonstrates the performance of the data rate of the network and the number of users. The number of users varies from five to 60 per BS; the number of BSs is set to 10; $P_{m}=10$ W; and $p_{c}=0.5$ W. One can notice that the total sum rate of the network is increasing with the number of users. Unlike the starting point, when the number of users reaches 30, the increasing of the sum rate becomes slower for all three NOMA-based methods. This is due to the efficient exploitation of the available spectrum. The increasing of the sum rate continues rapidly for OMA-FTPC until the number of users reaches 45, then becomes slower. The proposed method clearly outperforms NOMA-FTPA and OMA-FTPA. The performance of the proposed method is 2.7% better than NOMA-FTPA and 10.1% than OMA-FTPC. The DGA is 1.45% betterthan our proposed method because the objective function of the DGA is the sum rate maximization.

**Figure 5:**
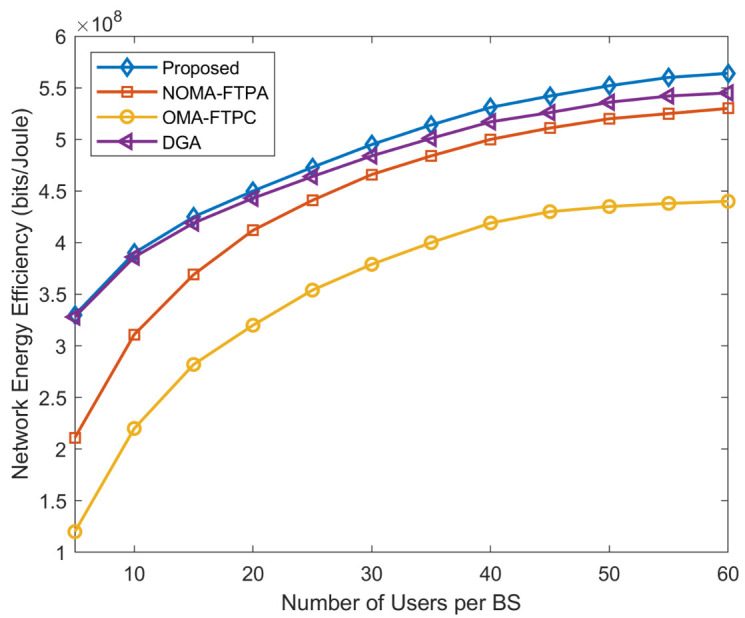
Network EE for different numbers of users per BS.

The EE of the network for the different number of users per BS is shown in Figure 5. It can be seen clearly that the EE of the network increases with the increasing number of users. The number of users varies from five to 60 per BS; the number of BSs was set to 10; $P_{m}=10$ W; $p_{c}=0.5$ W; and $R_{min}=0.1$ Mbits/s. We can also observe that the increment of EE is rapid for a lower number of users and rises slowly with a higher number of users. For example, when the number of users is between five and 30, the EE rises from $3.3\times 10^{8}$ bits/Joule to $4.95\times 10^{8}$ for the proposed method, from $3.28\times 10^{8}$ bits/Joule to $4.84\times 10^{8}$ bits/Joule for DGA, and from $2.11\times 10^{8}$ bits/Joule to $4.66\times 10^{8}$ bits/Joule for NOMA-FTPA.

**Figure 6:**
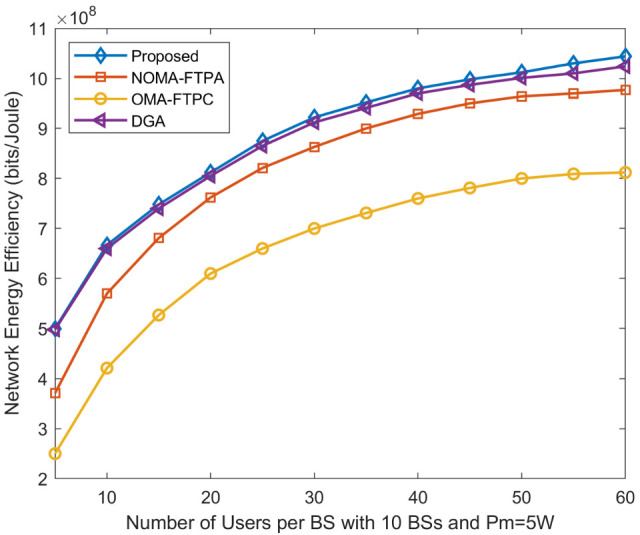
Network EE for different numbers of users per BS and P = 5 W.

However, for OMA-FTPC, the rapid growth of EE continues for a higher number of users. Generally, unlike the case of the sum rate, the performance of our proposed algorithm surpasses the DGA. Moreover, this difference in performance grows with the increase of the number of users, then it tends to saturate because the available power is not enough to achieve higher EE. A similar conclusion can be applied to Figure 6.

Figure 6 depicts the network EE of our proposed scheme and the performance comparison with other schemes for the different numbers of users per BS where the number of BSs is assumed to be 10 and the BS is 5 W, $p_{c}$ is 0.5 W, and $R_{min}=0.1$ Mbits/s. The proposed method outperforms other approaches followed by the DGA, NOMA-FTPA, and coming last, OMA-FTPC. Furthermore, it is worth mentioning that the performance difference between the proposed method and the DGA is increasing with the increasing of the number of users. When the number of users is 60, the proposed method is 2%, 6.4%, and 23.2% more energy efficient than the DGA, NOMA-FTPA, and OMA-FTPC, respectively.

**Figure 7:**
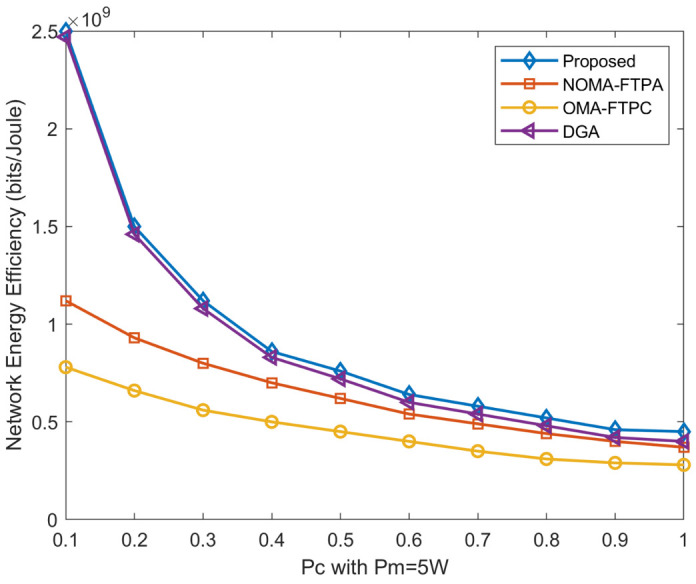
Network EE for different $p_{c}$ values.

Figure 7 represents the performance comparison of our proposed method with other methods with regard to network EE with different circuit power varying between 0.1 W and 1 W. It is obvious that the EE deteriorates with the increasing of circuit power consumption and $R_{min}=0.1$ Mbits/s. Therefore, reducing $p_{c}$ is one of the major goals in both device design and the research in the field information-theoretic and resource allocation. Nevertheless, it can be seen that the proposed method has the best performance among all methods. This superiority becomes clear with the growth of the circuit power consumption, especially in comparison with the DGA. When the circuit power is 0.5W, the proposed method has 6.25%, 15.6%, and 37.5% higher energy efficiency than the DGA, NOMA-FTPA, and OMA-FTPC, respectively.

## 5. Conclusions

In this paper, we investigate EE maximization in MCMC-NOMA networks. We formulate the problem as a non-convex NP-hard problem and investigate the solution of the problem. The proposed method begins with decomposing the problem into two subproblems, user association and power allocation. For user association, we design a matching-based algorithm to tackle this problem. For the second subproblem, the power allocation problem is still non-convex. We perform nested FP by quadratic transform to recast it into our power allocation problem to restore the convexity of our problem. The power allocation is obtained via iteratively solving the convex problem. The simulation results show that the proposed method has superior performance over other existing NOMA-based approaches, namely the DGA, FTPA, and OMA-FTPC. In the future, our work will include the investigation of the EE maximization in MIMO-NOMA networks.

## Figures and Tables

**Table 1:** Notations.

*M*	number of BSs
*N*	number of users
*K*	number of subcarriers
M	set of all cells
N	set of all users
K	set of all subcarriers
*W*	the total bandwidth of the system
$W_{k}$	bandwidth of the subcarrier
$p_{m,i,k}$	transmit power per user *i* associated with BS *m* through subcarrier *k*
$H_{m,i,k}$	channel response-to-noise and interference ratio
$\Gamma_{m,i,k}$	SINR
AU	list of associated users
UU	list of unassociated users
UB	list of preferable BSs
π	maximal EE
MU	list of matched users
UMU	list of unmatched users
UK	list of user’s preferable subcarriers
$T_{u}$	a tuple of matched user and the BS
$\varsigma_{m,i,k}$	an auxiliary variable associated with the single ratio quadratic transform
$y_{m,i,k}$	an auxiliary variable associated with the multidimensional quadratic transform
$R_{q}^{CF}$	an equivalent data rate function yielded from the closed-form FP and multidimensional quadratic transform
R	the set of real numbers
ϵ	the error tolerance

**Table 2:** Simulation parameters.

Parameter	Value
Overall bandwidth	5 MHz
Cell radius	500 m
Path loss	128.1+37.6$\log_{10}d$ dB, *d* is in km
Users distribution scheme	Randomly uniform distribution
Shadowing	Log-normal, standard deviation 8 dB
Fading	Rayleigh fading with variance 1
Noise power spectral density	−174 dBm/Hz
Number of users N	10 to 60 per BS
ϵ	0.001

## Abbreviations

The following abbreviations are used in this manuscript:

NOMA	Non-orthogonal multiple access
MCMC	Multi-cell multi-carrier
OMA	Orthogonal multiple access
FP	Fractional programming
1G	First-generation
4G	Fourth-generation
5G	Fifth-generation
SIC	Successive interference cancellation
OFDMA	Orthogonal frequency division multiple access
EE	Energy efficiency
MC	Multi-carrier
SCA	Successive convex approximation
ICI	Inter-cell interference
MOSs	Mean opinion scores
QoS	Quality of service
BS	Base station
SINR	Signal-to-interference-plus-noise ratio
MINLP	Mixed-integer non-linear programing
FTPA	Fractional transmit power allocation
FTPC	Fractional transmit power control
DGA	Double gradient algorithm
CSI	Channel state information
